# Benefits and risks of complete denture therapy in fully edentulous Indian patients

**DOI:** 10.6026/973206300200551

**Published:** 2024-05-31

**Authors:** Diptesh S Rami, Bhagyashree S Sutaria, Vishal B Parmar, Kishan Detroja, Rajesh Sethuraman, Trupti Makwana

**Affiliations:** 1 Department of Prosthodontics, Crown & Bridge, AMC Dental College and Hospital, Khokhara, Ahmedabad, Gujarat, India; 2 Department of Prosthodontics, Crown and Bridge, Siddhpur Dental College and Hospital, Dethli, Siddhpur, Gujarat, India; 3 Department of Prosthodontics and Crown & Bridge, Narsinhbhai Patel Dental College and Hospital, Visnagar, Gujarat, India; 4 Department of Prosthodontics and Crown & Bridge, College of Dental Scienceand Hospital, Amargadh, Bhavnagar, Gujarat, India; 5 Department of Prosthodontics, Crown & Bridge, K. M. Shah Dental College & Hospital, Sumandeep Vidyapeeth, Vadodara, Gujarat, India; 6 Consultant Prosthodontist, Anand, Gujarat, 388001, India

**Keywords:** Complete denture, completely edentulous patient, perception, benefit, risk

## Abstract

The perceptions of the patients regarding the benefits and risk of complete denture treatment in completely edentulous patients are of interest. The study composed of 79 participants who presented themselves for complete denture therapy. The perception of the participants towards the complete denture therapy was recorded using a validated questionnaire. The answers of the questionnaire were evaluated in three domains: (1) Benefits (positive perception) of the complete denture therapy' (2) Risks (Negative perception) of the complete denture therapy and (3) Consequences of no treatment. The data were tabulated for descriptive analysis. The average age of the total population was 62.22 ranging from 58 to 80 years. Total population included in the study shows positive perception regarding the complete denture therapy. The benefits of the complete denture therapy show highest score (1817) by all the participants while the risks of the complete denture therapy show lowest score (237). The gender, denture experience and the socioeconomic status had no influence on the perception towards the complete denture therapy. Participants showed positive perception towards the complete denture therapy with highest score in benefits and lowest score for the risks of the complete denture therapy. The perception of the complete denture therapy was not influenced by the gender, denture experience and the socioeconomic status.

## Background:

Edentulism is recognized as a hindrance that significantly affects the quality of life and nutritional well-being of individuals. Internationally, considerable numbers of those who are edentulous, particularly within the elderly demographic, stand in requirement of comprehensive rehabilitation. Demographic data pertaining to the aging population underscore the persistent and substantial demand for the rehabilitation of edentulous patients over the course of several decades. [1] Edentulous patients can have problems with feeding, speaking and socializing. Some edentulous patients may present an increase in psychological and social problems due to this handicap. Physical consequences of tooth loss also exist like residual ridge resorption, loss of facial support and a decrease in bite force and masticatory efficiency. [2] In the present era, a plethora of options exists for addressing the issue of missing teeth. Nevertheless, complete denture therapy persists as the cornerstone treatment for Edentulism and remains the preferred choice in numerous countries. This preference is attributed to its cost-effectiveness, aesthetic allure, and straightforward maintenance. Notably, the success of a complete denture has frequently relied upon the clinical judgment of the dentist, who evaluates crucial attributes such as retention, stability, aesthetics, and occlusion. [3- 4, 5] Epidemiological investigations into Edentulism and tooth loss exhibit significant variations, manifesting notable differences in prevalence not only between countries but also within specific geographic regions and among diverse patient groups with varying backgrounds. Certain studies have highlighted a correlation between the incidence of Edentulism and educational levels, as well as income status. Notably, individuals in lower educational and income brackets tend to exhibit higher risks of complete tooth loss. Conversely, other studies, such as those referenced in, indicate an escalating number of elderly individuals retaining their natural dentition, thereby presenting the challenge of increased demands for dental care. [6, 7] The effectiveness of traditional complete denture therapy may be influenced by a myriad of factors. These include the patient's age, personality; prior experience with dentures, expectations, aesthetic preferences, residual ridge form and anatomy, the quality of the denture, the methodology employed in its construction, the experience of the dentist and the dynamics within the dentist-patient relationship. [8, 9] Notably, patient satisfaction emerges as a pivotal factor in this equation. By adhering to precise diagnostic procedures and stringent protocols, achieving and maintaining high levels of patient satisfaction becomes instrumental in securing enduring clinical outcomes over the long term. [10] Traditional complete dentures continue to be the favored therapeutic approach for individuals without teeth, demonstrating an enhancement in oral health-related quality of life. [11,12] Widely embraced, complete dentures not only fulfill aesthetic expectations but also facilitate regular speech, while offering essential occlusal support for effective chewing. It is imperative that these dentures not only meet the criteria of comfort but also yield high levels of patient satisfaction, both of which stand as primary objectives in the realm of treatment. [13] It is imperative for the dentist to comprehend the expectations of patients concerning prosthetic therapy. This understanding is crucial for elucidating the genuine possibilities of the treatment. By doing so, the dentist can preemptively mitigate potential frustration stemming from unrealistic expectations, fostering a constructive relationship with the patient. This approach not only aids in determining the realism of expectations in a given scenario but also provides insight into whether conventional Prosthodontic procedures are likely to meet these expectations. [14] Therefore, it is of interest to assess the patients' perspectives on the advantages and potential risks associated with complete denture treatment in individuals who are fully edentulous.

## Materials and Method:

A study was conducted to evaluate the patients' perception on the advantages and risks associated with complete denture therapy through a questionnaire-based approach. Ethical clearance for the study was secured from the institutional ethical committee, granted under approval number SVIEC/ON/DENT/SRP/16027.The study was conducted in the Department of Prosthodontics at Sumandeep Vidyapeeth Deemed to be University, Vadodara, Gujarat, India. Based on the reference values reported in the study by Miranda BB *et al.* 2, a sample size of 79 was determined using a specific formula. The patients seeking treatment for complete dentures at the Department of Prosthodontic, Crown and Bridge underwent screening for potential inclusion in the study. Information about the study will be conveyed to them through the Participant Information Sheet and their participation will be contingent upon providing Informed Consent. The inclusion criteria for participant selection in this study encompass individuals who are completely edentulous. Individuals who decline to provide informed consent for participation in the study, patients with implants or those opting for dentures rehabilitated with implants and individuals presenting with sub mucous fibrosis or other mucosal conditions were excluded.

## Evaluation of clinical and patient-related factors:

At the initiation of complete denture therapy, pertinent clinical variables and patient-related details including gender, age and educational level were meticulously recorded in the patient records. Furthermore, the evaluation encompassed an examination of the socioeconomic status of the participants; a vital aspect assessed using Kuppuswamy's Socio-Economic Status Scale.[15]

## Assessment of patients' perception of complete denture therapy

The assessment of patients' perceptions regarding their individual complete denture therapy utilized a questionnaire adapted from the one proposed by Leles *et al.* [16] This questionnaire was modified to align with the specific circumstances of edentulous patients. To ensure linguistic and cultural appropriateness, the questionnaire was translated into Gujarati using a translation-back translation method. The translated questionnaire underwent validation before its utilization in the study (Annexure 1). Each question in the questionnaire was assigned a score based on a Likert-type scale, ranging from 01 (totally disagree) to 05 (totally agree). The responses were categorized into three domains: Benefits (reflecting positive perceptions), Risks (indicating negative perceptions), and Consequences of no treatment.

The questionnaire was personally administered at the department's outpatient department or during the course of treatment. Patients were provided with the questionnaire form to fill out. Subsequently, the evaluation of the questionnaire involved four scores: one for the entire questionnaire and three specifics to the consequences of no treatment, risk, and benefit domains. Descriptive statistics were employed to analyze the scores obtained. Additionally, the impact of variables such as gender, denture experience, and socioeconomic status on the perception of benefits and risks associated with complete denture therapy in completely edentulous patients was assessed using the Chi-square test.

## Results:

**Figure 1:**
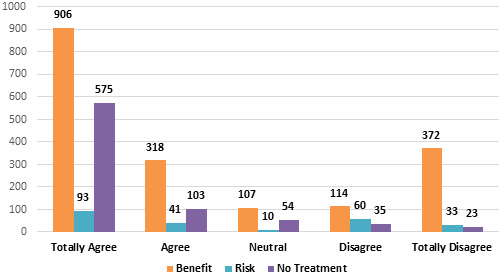
Frequency Distribution according to perception towards complete denture therapy

As shown in Table 1, out of 79 participants, fifty-six were male and twenty-three were female and average age of entire sample was 62.22 ranging from 58 to 80 years. Table 1 also shows that fifty-two participants were new denture wearer and twenty-seven were old denture wearers. Table 2 shows that According to Kuppuswamy's Socio-Economic Status Scale10, all the participants were divided in five groups. According to that the participants were scored and categorized. Upper class (1), Upper Middle class (2), Lower Middle class (3), Upper Lower class (4) and Lower class (5). Among all participants, only 1 participant was fit in the Upper class. Seven were in Upper middle class, 15 were in Lower Middle class, 53 were in Upper Lower class and 3 were in Lower class. The majority of the participants were in the Upper Lower class. Regarding the perception of complete denture therapy, the entire participant was positive about the complete denture therapy. The Benefits of complete Denture therapy received highest score followed by consequences of no treatment and risk factors which received lowest score (Table 3 and Figure 1).

The Table 4 shows a comprehensive overview of the association between questionnaire responses and key demographic factors. Examining the gender factor reveals interesting patterns. In general, there seems to be a balanced distribution of responses between males and females across the questions. Chi-square tests suggest that the observed gender differences are not statistically significant in most cases, with p-values exceeding conventional significance levels (e.g., 0.05) except for response to Q12. This indicates that the variations observed could be due to random chance rather than a genuine association between gender and responses. The denture experience factor, categorized into 'Old' and 'New,' is another intriguing aspect. The analysis indicates that responses do not significantly differ between individuals with old denture experiences and those with new experiences. The p-values for most comparisons are higher than typical significance thresholds, suggesting that denture experience may not strongly influence responses except in Q24 and Q25. The socioeconomic status factor, divided into various classes, demonstrates more noticeable patterns. This suggests that individuals from different socioeconomic classes may have varying opinions or experiences related to the questionnaire. Questions 15, 24, and 25 also exhibit significant associations, hinting at potential socioeconomic disparities in specific aspects addressed by these questions.

## Discussion:

Patients' satisfaction with complete denture rehabilitations is a complex and multifaceted phenomenon influenced by a myriad of individual characteristics, psychological factors, the adaptation process and perceived health needs. These factors must be carefully considered within the broader socio-cultural and economic context to provide effective and patient-centered dental care. Dental professionals play a crucial role in understanding and addressing these variables to enhance the overall patient experience. [17] Studies have found that neurotic patients are less satisfied with their dentures. Patients who are well-satisfied with their daily life are also satisfied with their dentures. Positive affects like joy, peace and usefulness can cause denture acceptance, while negative affects like boredom, anger, loneliness and helplessness can cause denture intolerance. [18] Factors such as age, gender, level of education and self-perception of affective and economic status can influence patient satisfaction with complete dentures. Patients with a higher level of education and better self-perception of their affective status show higher satisfaction, while patients with a higher self-perception of their economic status show lower satisfaction. [19, 20]

The null hypothesis of this study posited that gender, educational level, and previous denture experience would not exert any influence on the perceptions of complete denture therapy. The analysis of the data suggests that the null hypothesis has been supported, indicating that these demographic and experiential factors did not have a statistically significant impact on patients' perceptions of complete denture therapy. In other words, patients generally exhibited positive perceptions toward complete denture therapy and variations in gender, denture experience and socioeconomic status did not yield significant differences in these perceptions.

Satisfaction of the denture wearers decreased with increase in duration since the patients are wearing denture as quoted in the study by Subramanian *et al.* [10]. The present study also shows that the perception of the people was found to be significantly associated whether they were first wearers or old wearers of denture. Furthermore, psychological status of patients wearing denture is affected during the Prosthodontic treatment and establishes the need to include the psychosomatic component in the Prosthodontic treatment. [21] In similar lines, study by Shah R J *et al* also explored the association between the emotional reactions to tooth loss and any depressive symptoms. The study revealed a significant association between tooth loss and depressive symptoms. [22] Improvement in oral health related quality of life is affected by the psychological status of the edentulous patients. [23]

Notably, despite the overall positive perception, the domain of risk perception presented the lowest scores among the questionnaire domains. While patients might express slightly lower scores in the domain of risk perception, these variations were not substantial enough to be deemed statistically significant. This outcome aligns with findings from a prior study, which similarly did not identify statistically significant differences in the perception of potential risks or negative views about removable dentures. [2, 16] Therefore, the current study's results are consistent with existing literature, suggesting a general trend where demographic factors and previous experiences do not strongly influence patients' overall perceptions of the risks associated with complete denture therapy. In essence, this study contributes to the understanding that patients' attitudes and perceptions towards complete denture therapy are relatively consistent across diverse demographic and experiential categories. The lack of significant differences in perceptions underscores the need for a holistic and patient-centered approach in dental care, acknowledging that individualized factors may not be strong determinants of how patients perceive and respond to complete denture therapy. This knowledge can guide dental professionals in providing tailored and effective communication about the therapy, with a focus on addressing individual concerns and enhancing overall patient satisfaction.

## Conclusion:

Data shows no statistically significant influence of gender, educational level, or previous denture experience on patients' perceptions of complete denture therapy. The study underscores the importance of a holistic, patient-centered approach in dental care, acknowledging that demographic factors may not strongly determine how patients view complete denture therapy, while statistically insignificant; the variations in risk perception scores highlight the need for dental professionals to address and communicate potential challenges associated with complete denture therapy. Further research and ongoing efforts to understand patient perspectives will continually refine dental practices, ensuring quality care for diverse patient populations.

## Figures and Tables

**Table 1:** Frequency Distribution of Participants

**Gender**		**Denture experience**	
		**Old**	**New**
Male	56	18	38
Female	23	9	14
Total	79	27	52

**Table 2:** Frequency distribution according to socioeconomic status

**Gender**	**Upper class(U)**	**Upper Middle class(UM)**	**Lower Middle class(LM)**	**Upper Lower class(UL)**	**Lower class(L)**
Male	1	5	12	38	0
Female	0	2	3	15	3
Total	1	7	15	53	3

**Table 3:** Frequency Distribution according to perception towards complete denture therapy

	**Benefit**	**Risk**	**No Treatment**
Totally Agree(TA)	906	93	575
Agree(A)	318	41	103
Neutral(N)	107	10	54
Disagree(D)	114	60	35
Totally Disagree(TD)	372	33	23
Total	1817	237	790

**Table 4:** Association of Significant Responses to the questions with gender, denture experience and socio-economic status

**Ques.**	**Factors**		**TD**	**D**	**N**	**A**	**TA**	**p value**	**Chi Square test**
12	Gender	M	0	1	4	24	27	0.034	8.676
		F	0	0	7	10	6		
15	Socioeconomic Status	U	0	0	0	0	1	0.05	25.776
		UM	2	1	0	3	1		
		LM	8	4	0	2	1		
		UL	12	20	1	18	2		
		L	0	2	0	1	0		
24	Denture Experience	Old	11	11	0	3	2	0.014	12.533
		New	7	29	10	3	3		
		Female	6	4	0	10	3		
25	Denture Experience	Old	10	5	0	9	3	0.026	9.291
		New	5	13	0	21	13		
27	Socioeconomic Status	U	0	0	0	1	0	0.001	25.363
		UM	0	0	0	1	6		
		LM	1	0	0	0	14		
		UL	0	0	0	2	51		
		L	0	0	0	0	3		
TA: Totally Agree, A: Agree, N: Neutral, D: Disagree,
TD: Totally Disagree; U: Upper Class, UM: Upper Middle Class,
LM: Lower Middle Class, UL: Upper Lower Class, L: Lower Class
